# Localization of deformed wing virus infection in queen and drone *Apis mellifera *L

**DOI:** 10.1186/1743-422X-3-16

**Published:** 2006-03-28

**Authors:** Julie Fievet, Diana Tentcheva, Laurent Gauthier, Joachim de Miranda, François Cousserans, Marc Edouard Colin, Max Bergoin

**Affiliations:** 1 Laboratoire de Pathologie Comparée des Invertébrés EPHE, UMR 1231 Biologie Intégrative et Virologie des Insectes INRA, Université Montpellier II, Place Bataillon, 34095 Montpellier, France; 2 Department of Entomology, Penn State University, PA16802, USA

## Abstract

The distribution of deformed wing virus infection within the honey bee reproductive castes (queens, drones) was investigated by *in situ *hybridization and immunohistology from paraffin embedded sections. Digoxygenin or CY5.5 fluorochrome end-labelled nucleotide probes hybridizing to the 3' portion of the DWV genome were used to identify DWV RNA, while a monospecific antibody to the DWV-VP1 structural protein was used to identify viral proteins and particles. The histological data were confirmed by quantitative RT-PCR of dissected organs. Results showed that DWV infection is not restricted to the digestive tract of the bee but spread in the whole body, including queen ovaries, queen fat body and drone seminal vesicles.

## Findings

More than fifteen viruses have been described from honey bees (*Apis mellifera *L.) to date, most of which are 30 nm isometric particles containing a single positive strand RNA genome [1]. These viruses are widespread in honey bee colonies [2,3] with multiple virus infections in the same bee colony a common feature [3-7]. These infections are generally low level and symptomless [4,8], with occasional outbreaks producing clinical signs at individual bee or colony level [1]. Many infected bees remain asymptomatic and functional, although usually with a reduced life span [9]. This relatively benign scenario changed with the arrival of *Varroa destructor *which activates and transmits several of these viruses, resulting in greatly elevated incidence of these viruses [1,3,10]. Of these, deformed wing virus (DWV) appears to be closely associated with *Varroa destructor *infestation of bee colonies [11-14].

Queen fecundity is a central element in colony performance for honey production that could be impaired by viral infections [6,15]. For instance, the undesired queen supersedure observed regularly by beekeepers may be related to viral infections. There are several reasons for untimely queen changing by workers in a colony, such as pathological impairment of its reproductive functions, lack of pheromone emission and lack of fully active spermatozoa in the spermatheca and decreasing sperm viability with the ageing of queens [16]. Very few investigations have been published regarding factors affecting the fertility of the queens and the drones [17].

To study more precisely the etiology of DWV infection and to identify pathological effects on bee reproduction, we have attempted to localize DWV nucleic acid and viral particles in queen and drone organs by *in situ *hybridization and immunohistology. In parallel, tissue samples were analyzed by quantitative PCR to estimate the number of viral genome copies in the organs.

DWV was detected by triplicate quantitative RT-PCR assays [14] in 67% of asymptomatic egg laying queens (n = 83), in 78% of drones collected at emergence (n = 14) and in 100% of drones collected at hive entrance (n = 12). For absolute quantification of DWV genome copies, calibration curves were established from a tenfold diluted DWV PCR fragment as described [14]. The viral loads recorded in samples varied from 10^6 ^to 10^12 ^DWV genome equivalent copies (DWV-geq) per bee, with no statistical difference between queens or drones as determined by analysis of variance on ranks (p = 0.88). Several healthy and infected queens and drones were dissected and their organs were extensively washed before quantitative RT-PCR analysis. In drones, the highest DWV RNA loads were recorded in testis (1.1 × 10^9 ^DWV-geq) and in the digestive tract (1.5 × 10^9 ^DWV-geq) followed by mucus glands (1.5 × 10^8 ^DWV-geq) and seminal vesicles (9.0 × 10^7 ^DWV-geq). DWV was also detected in the head (2.7 × 10^6 ^DWV-geq) and in sperm (4.7 × 10^2 ^DWV-geq). In queens the ovaries had the largest DWV RNA load (3.2 × 10^7 ^DWV-geq) followed by the head (2.5 × 10^5 ^DWV-geq) and digestive tract (1.0 × 10^5 ^DWV-geq).

The *in situ *localization of DWV infection in bee tissues was done according to [18], except that samples were fixed in 4% paraformaldehyde in PBS at 4°C for 24 hours. Paraffin-embedded tissue sections were either challenged with a monospecific rabbit polyclonal antibody raised against, and shown to react exclusively with, the DWV VP1 protein [19] or with the following oligonucleotide probes at a concentration of 200 pmol/ml:

- DWVantisense: 5'-TACTGTCGAAACGGTATGGTAAACTGTAC-Digoxygenin

- DWVsense: 5'-GTACAGTTTACCATACCGTTTCGACAGTA-Digoxygenin

- DWVnonsense: 5'-CATGTCAAATGGTATGGCAAAGCTGTCAT-Digoxygenin

The antisense probe hybridizes in the DWV RNA polymerase RNA dependent domain while the homologous sequences (sense and nonsense probes) were used in parallel as controls. Serological and hybridization events were detected by incubating the sections with goat anti-rabbit IgG antibody (Tebu) or anti-digoxygenin antibody (Roche) respectively, both conjugated to alkaline phosphatase, and developed with nitroblue tetrazolium and 5-bromo 4-chloro 3-indolyl phosphate [20]. For laser scanning microscopy, the antisense oligonucleotide probe was 5' labeled with the fluorochrome Cy5.5 (MGW Inc.) and used at 200 pmol/ml; a control was performed in parallel using 20 nmol/ml of unlabelled antisense probe as competitor.

**Figure 1:**
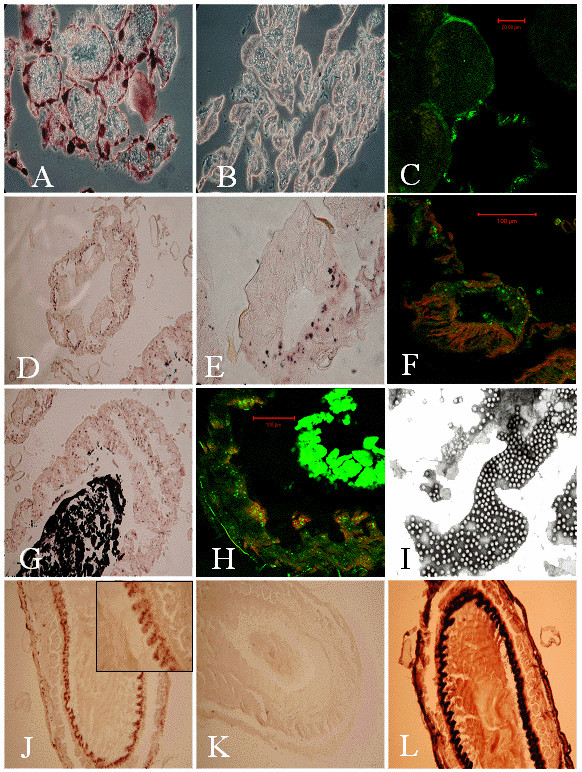
Detection of DWV by *in situ *hybridization (A to K) and immuno-histochemistry (L) in queen and drone organs. A, B, D, E, G, J, K, and L: Light microscopy. C, F and H: Laser scanning microscopy (CY5.5 fluorochrome specific signal is in green while autofluorescent background is in red; observed on a Zeiss LSM510 META laser scanning microscope). I: Electron microscopy. A-C: Queen fat body challenged with digoxygenin labeled anti-sense (A) and sense (B) probes and with CY5.5 labeled antisense probe (C). D – F: Drone rectal pads challenged with digoxygenin (D, E) and CY5.5 (F) labeled antisense probes. E and F: detail of a rectal pad (from D). G and H: Drone midgut challenged with digoxygenin (G) and CY5.5 (H) labeled anti-sense probes. I: Electron microscopy analysis of drone midgut content (crude extract). J – L: Drone seminal vesicle challenged with anti-sense probe (J), sense probe (K) and with anti DWV-VP1 polyclonal antiserum (L). Square in J: detail of the signal obtained with digoxygenin labeled anti-sense probes on internal drone seminal vesicle mucosa.

Our attempts to localize DWV in the queen digestive tract and in ovaries using both *in situ *hybridization and immunohistochemistry were unsuccessful despite the presence of DWV RNA revealed by quantitative RT-PCR. However, a strong and specific detection was observed in queen fat body cells (Figure 1A and 1C; sense negative control in B). The signal was clearly restricted to cytoplasm and plasma membrane of a majority of fat body cells, as shown by light microscopy (Figure 1A) and laser scanning microscopy (Figure 1C). In insects, this organ fulfils a series of essential metabolic and endocrine functions in addition to its important role in food storage. It is also the site of production of many antimicrobial peptides [21]. Thus, viral infection of fat body cells may impair insect development and physiology and may lead for example to immuno-suppression, an effect so far attributed mainly to varroa mite parasitism [22]. In queens, the fat body cells produce vitellogenin, the yolk protein accumulated during egg maturation. Thus, DWV infection of queen adipose cells might also impair egg production.

In drones, a strong DWV specific response was observed in the digestive tract (Figure 1D–H). The virus was detected in a majority of epithelial cells located in the proventriculus, midgut and hindgut. In the latter, the infection was confined to the cells corresponding to the external wall of the rectal pads with no DWV detected in the longitudinal cells forming the inner wall (Figure 1D–F). In the midgut epithelium, the virus was clearly detected in most of the mature columnar cells suggesting that the digestive process could be significantly impaired by the infection. The midgut content was full of mature virus particles (Figure 1I) which reacted strongly with the DWV specific antisense probe (Figure 1G and 1H). In the drone reproductive tract, DWV was detected in most of the tissues, especially in the seminal vesicles where the whole internal epithelium was clearly stained with both the DWV-VP1 antibody and the antisense probe (Figure 1J and 1L; sense negative control in K). These cells play an important role in spermatozoa maturation. Intensive replication of DWV in this tissue could therefore have a negative effect on drone fertility. The mucus glands and testis epithelia were also shown to be infected. The presence of DWV in these tissues explains the detection of DWV RNA in the sperm, through which drones could contaminate queens and the next generation's worker brood following fertilization. This interpretation is indirectly supported by previous results showing a decrease in flight performance and sperm production in drones parasitized by *V. destructor*, an efficient vector of DWV [17].

These data show that DWV infection has a considerable degree of tissue specificity. The distribution and accumulation of viruses in different honeybee tissues has also been determined previously by ELISA for acute bee paralysis virus (ABPV) and slow paralysis virus (SPV), two other viruses associated with varroa infestation. ABPV accumulated almost exclusively in the hypopharyngeal, mandibular and salivary glands, with minor accumulation in the crop, midgut and hind legs, while SPV had a slightly wider distribution, accumulating also in the brain and fat body [23]. With the exception of these data, the main data currently available on virus localization in honey bee tissues were obtained using non specific methods such as classical histological staining methods [24] and electron microscopy [25-31]. Here we show that several bee tissues can be infected by DWV, particularly in the digestive and the reproductive organs. Many epithelia are enclosed by a basal lamina which constitutes a physical barrier against viral particles and hence a protection of the internal tissues against infection. This may explain the striking difference in infection efficiency and virulence between oral and mite-mediated DWV transmission, since by piercing the mite can easily by-pass these protective barriers, delivering the virus directly to the developing bee organs during the pupal phase. Such infection is far more difficult to achieve through trophallaxis between adults or through oral infection of bee larvae by nurse bees. It is also noteworthy that some viruses are able to cross this lamina through tracheal cells [32] and through micro abrasions caused by direct contact between individuals [1].

The concentration of DWV in the reproductive tissues of both queens and drones suggests that DWV infection could have deleterious effect on their reproductive fitness, which would seriously affect colony performance and productivity, swarming and queen supercedure. The DWV presence in sperm implies a possible sexual transmission route for this virus, which could have major implications for virus transmission between colonies [33] and queen rearing operations.

## Abbreviations

RT-PCR: reverse transcriptase polymerase chain reaction

## Competing interests

The author(s) declare that they have no competing interests.

## Authors' contributions

JF and DT contributed equally to this work. JF did the *in situ *hybridization experiments. DT performed the quantitative PCR experiments. LG planed the experiments and wrote the manuscript. JdM, FC, MEC and MB contributed to the design of the experiments and revised critically the manuscript. All authors read and approved the final manuscript.
